# Complex intervention programme to improve patient safety and facilitate deprescribing in frail older patients living at home (COFRAIL): A process evaluation of a cluster randomised controlled trial

**DOI:** 10.1371/journal.pone.0350664

**Published:** 2026-07-08

**Authors:** Jens Abraham, Steffen Fleischer, Achim Mortsiefer, Eva Drewelow, Manuela Ritzke, Birgitt Wiese, Charalabos Markos Dintsios, Veronika Bencheva, Petra Thürmann, Stefan Wilm, Gabriele Meyer, on behalf of the COFRAIL study group

**Affiliations:** 1 Institute of Health, Midwifery and Nursing Science, Medical Faculty of Martin Luther University Halle-Wittenberg, Halle (Saale), Germany; 2 Faculty of Health, Department of Medicine, Institute of General Practice and Primary Care, Witten/Herdecke University, Witten, Germany; 3 Clinic for Forensic Psychiatry, University Medical Centre Rostock, Rostock, Germany; 4 Institute of General Practice, University Medical Centre Rostock, Rostock, Germany; 5 WG Medical Statistics and IT-Infrastructure, Institute of General Practice, Hannover Medical School, Hannover, Germany; 6 Faculty of Medicine, Institute for Health Services Research and Health Economics, Centre for Health and Society, Heinrich-Heine-University Düsseldorf, Düsseldorf, Germany; 7 Faculty of Health, Center for Clinical Trials, Witten/Herdecke University, Witten, Germany; 8 Chair of Clinical Pharmacology, Department of Medicine, Faculty of Health, Witten/Herdecke University, Witten, Germany; 9 Philipp Klee-Institute of Clinical Pharmacology, Helios University Hospital Wuppertal, Wuppertal, Germany; 10 Institute of General Practice, Medical Faculty, Heinrich-Heine-University Düsseldorf, Düsseldorf, Germany; University of Toronto, CANADA

## Abstract

**Introduction:**

Frailty is associated with negative health outcomes in geriatric patients. A large proportion of frail patients is affected by polypharmacy, which in turn may be a possible cause of frailty. The cluster randomised controlled COFRAIL trial investigated the effects of family conferences to improve the care of frail patients. Deprescribing and communication about prioritising health goals between patients, relatives and general practitioners were key components of the intervention. An accompanying process evaluation was conducted to investigate whether the study intervention was implemented as intended and to describe the experiences of the target groups.

**Methods:**

The process evaluation took place between February 2019 and October 2021 and followed international guidelines. Process parameters were collected using study documentation, standardised questionnaires and guided telephone interviews with a convenience sample of patients, relatives and general practitioners. Quantitative data were analysed descriptively, qualitative data through content analysis.

**Results:**

Almost all general practitioners in the intervention group completed both mandatory trainings. Overall, 68% of the patients took part in all three planned family conferences, 85% in at least two. Patients, relatives and general practitioners reported positive experiences with the family conferences. Patients and relatives felt involved in the decision-making process and had predominantly no concerns when medication was discontinued. Only a few general practitioners considered the implementation of family conferences in regular care to be impractical. A supportive pharmacological hotline was not utilised frequently by the general practitioners. Due to the SARS-CoV-2 pandemic, some study procedures were adapted but did not have any negative impact.

**Conclusions:**

While the COFRAIL study did not achieve its primary goals, we identified no major barriers in the implementation of the intervention programme. Overall, the target groups experienced the approach of family conferences positively. However, the process evaluation provides valuable lessons for designing and implementing similar interventions in the future.

## Introduction

The frailty syndrome is a complex problematic health condition that is common in the older population. It is characterised by decreased physiological reserves and vulnerability to stressors [1–3]. Data on the prevalence of frailty vary widely, as studies are based on different operationalisations of frailty status [4]. A recent meta-analysis [5] with 57 studies and a total of 56,407 older people (mean age 78.6 years) found an overall prevalence of 26.8% and of 13.3% in community-based studies using a multidimensional approach for the definition of frailty (Multidimensional Prognostic Index (MPI) [6]).

An increased risk of falls, delirium, malnutrition, adverse drug events, inappropriate prescribing, hospitalisation as well as mortality is associated with this geriatric syndrome, especially in the context of polypharmacy [3,7–10]. A large proportion of older people is affected by polypharmacy, which in turn may cause or exacerbate frailty [1,11]. There is no consented definition of polypharmacy and a review identified 138 different definitions [12]. Most often, polypharmacy is defined as taking five or more drugs daily. The prevalence varies widely, not only depending on definitions that have been applied, but also due to the case mix of the population in terms of chronic conditions, demographic characteristics and the underlying setting [13,14].

The two-arm cluster randomised controlled trial (COFRAIL) [15,16] (trial registration: DRKS00015055) with a total of 113 general practitioners (GPs) in 110 practices (clusters) and 521 older, frail patients investigated the effectiveness of family conferences as an approach to improve the care of this target group. Identification of polypharmacy with jointly considered discontinuation of drugs (deprescribing) [17] and communication about prioritisation of health goals between patients, their relatives and/or the ambulatory care service and their GPs during family conferences were main components of this complex intervention. The deprescribing process is inherently complex and thus requires more effective communication between all involved interest-holders [18,19]. In community-dwelling older patients, family members frequently play a key role in managing medications [20–23]. Therefore, strategies aimed at deprescribing should be incorporated into a shared decision-making framework which actively involves GPs, patients and family members. Family conferences are a well-established instrument to improve communication in various healthcare settings [24–30]; their impact on drug safety in frail, older patients receiving home care was investigated for the first time in the COFRAIL study [15,16].

The GPs in the intervention group received a training programme over a period of six months. Two mandatory training sessions and a third optional training were provided. The training focused particularly on the topics of deprescribing and non-pharmacologic interventions, and included communication training to prepare the GPs for the structured family conferences. The patients in the intervention group, along with their family carers and/or the home care service, were offered three family conferences over a period of nine months, conducted either in the patient’s home or at the GP’s practice. The main focus of the family conferences was to review the current medication and deprescribe drugs if indicated, supported by a deprescribing manual [31], and, if needed, to consider further non-pharmacological interventions from a toolbox [32]. Furthermore, the GPs had the opportunity to get counselling from an external clinical pharmacologist/pharmacist via phone or email and to ask clinical and pharmaceutical questions regarding their study patients (pharmacological hotline). The participants of the control group received care as usual.

The COFRAIL intervention led to a significant decrease in the overall number of medications and the number of potentially inappropriate medications (PIM) after six months but did not reduce the number of hospitalisations, the number of medications, or the number of PIM compared to usual care after 12 months. The report on the effectiveness study and details about the development and content of the COFRAIL intervention including the underlying theoretical rationale are published elsewhere [15,16,32].

Due to the complex nature of the intervention and the study setting, a comprehensive process evaluation was carried out as recommended by the UK Medical Research Council’s (MRC) guidance on the development and evaluation of complex interventions [33]. According to the MRC, a strictly quantitative approach that relies solely on an experimental design, without incorporating elements like process evaluation, is rarely sufficient for researching complex interventions. A process evaluation enables understanding of fidelity and quality of an intervention’s implementation, mechanisms of change, and context. Moreover, it provides insights into why an intervention may fail unexpectedly and lead to unintended effects, or how the intervention might be improved or refined [33]. This paper reports on the process evaluation conducted alongside the COFRAIL study [15,16] and aims to evaluate the implementation of the intervention, including barriers and facilitators, as well as to explore the experiences of the participating target groups – patients, relatives and GPs.

## Materials and methods

### Design

The process evaluation was designed based on the recommendations for process evaluations for cluster randomised trials of complex interventions proposed by Grant et al. [34]. Different process parameters were collected at the cluster and individual level using various qualitative and quantitative methods. The process evaluation was carried out between 01st February 2019 and 31st October 2021.

### Setting

This study was performed in a primary care setting in two areas of Germany (Düsseldorf (North Rhine-Westphalia) and Rostock (Mecklenburg-Western Pomerania)). The process evaluation involved the participating GPs, patients and relatives of the intervention group.

### Data collection methods

To investigate the feasibility and optimise the intervention, semi-structured telephone interviews were conducted with GPs, patients and relatives after piloting the family conferences. The piloting has been reported elsewhere [32].

The recruitment process, including information provided on reasons for non-participation or drop-out, was documented at both the cluster and individual level. The results have been published together with the effectiveness data [15]. To determine intervention fidelity, structured documentation forms were completed by instructors after each mandatory educational session. Furthermore, the mandatory educational sessions were evaluated by participants after the second session using a standardised questionnaire (satisfaction with the training, attitudes towards deprescribing, acceptance of intervention, self-efficacy, expectations; rated by 4-point Likert scales (1 = strong agreement to 4 = strong disagreement)). For the third optional educational session, only the utilisation was documented. In addition, the family conferences were documented by the GPs using a semi-structured protocol immediately after each family conference. The utilisation of the individual medication reviews provided by a pharmacologist/pharmacist was also documented.

**Fig 1:**
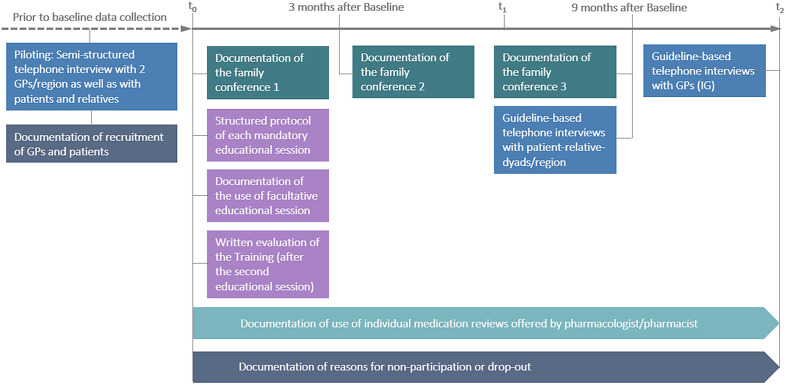
Timeline of the process evaluation procedures. Measurement points: t_0_=baseline COFRAIL study, t_1_=after 6 months, t_2_=after 12 months. Abbreviations: IG=intervention group; GP=general practitioner.

At the end of the study, the attitudes of GPs towards the COFRAIL intervention and the experiences, including barriers and facilitators, were assessed in guided telephone interviews. Guided telephone interviews were also conducted with patients and their relatives regarding families’ experiences (e.g., consideration of preferences, changes in physician-patient-communication, barriers and facilitating factors). The interviews were conducted by researchers not involved in the main study, and who had at least a Master`s degree in health and nursing science, and extensive experience in qualitative data collection. Both interview guides were pretested during the piloting phase of the COFRAIL study. The interview guides for the telephone interviews with GPs, patients and relatives are provided in additional S1 and S2 Appendix. Fig 1 illustrates the timeline of the process evaluation procedures. A comprehensive overview of the focus, methods and target groups of the process evaluation can be found in additional file S3 Appendix.

### Sample

For the evaluation of the mandatory educational sessions, all participating GPs were asked to complete the evaluation questionnaires after the second session. The guideline-based telephone interviews with GPs at the end of the study were conducted with a convenience sample (all GPs willing to participate were included, with no specific sampling criteria applied) of at least ten GPs per region. At the end of the mandatory training sessions, the GPs were asked about potential interest in further interviews and were later invited to participate. No incentives were offered.

Initially, it was planned to conduct guideline-based telephone interviews with a convenience subsample (interested to participate, with no specific sampling criteria applied) of ten patient-relative-dyads per region. Since the relatives could not be reached through patients in many cases or joint interview appointments with relatives and patients were not feasible, the telephone interviews were mostly conducted separately with either patients or relatives. For this purpose, the study nurses at the two study centres in Düsseldorf and Rostock inquired about potential interest among eligible patients and relatives during the main data collection and, if necessary, sent the contact data to the study centre in Halle (Saale) for process evaluation. Subsequently, a written invitation to participate in the interview was sent out. Participants had to be physically and mentally able to take part in a telephone interview. An exclusion criterion of the COFRAIL study and therefore of its process evaluation was the inadequate German language proficiency of the patient and family caregivers, or the unavailability of an interpreter. There were no further inclusion or exclusion criteria.

### Ethics

The COFRAIL study, including the process evaluation, was approved by the ethics committees of the study centres Rostock (no. A2018-0151) and Düsseldorf (no. 2018−283). Written informed consent was obtained from all interview participants. Participants in the evaluation of the educational programme gave their consent by returning the questionnaires.

### Data analysis

The process evaluation was exploratory. The quantitative data were analysed descriptively, calculating mean values, standard deviation, minimum – maximum, absolute and relative frequencies. The qualitative data were analysed according to Maying’s qualitative content analysis [35]. All interviews were digitally audio recorded and transcribed verbatim based on the transcription rules by Dresing & Pehl [36]. The initial themes were coded deductively by one person (JA) using a set of categories derived from the research question and the interview guides applied to each interview. Based on the interview material, additional themes were developed inductively. Finally, findings were discussed and interpreted with another member of the study team (SF). The contents were summarised and narratively described. MAXQDA software (version 2020) was used for the analysis. The interviews were held in German, and participants’ quotes were translated into English by the authors (JA & SF) and double-checked by a native speaker.

## Results

### Implementation of educational programme

A total of 26 first training sessions, 21 second training sessions -both mandatory- and three third training sessions (optional) were provided. The first training lasted on average 137 minutes (median: 120 minutes; SD: 42.9; min: 70 minutes; max: 210 minutes), the second training 103 minutes (median: 90 minutes; SD: 31.6; min: 60 minutes; max: 195 minutes). Data on the duration of the third training is not available.

All 59 GPs (from 56 intervention practices) attended the first training. Only one GP was unable to take part in the second training session, but all the training materials were provided. A total of 26 GPs participated in optional training session three. Due to the SARS-CoV-2 pandemic, all training sessions were held online in groups or individually by telephone from March 2020 onwards (training session 1: n = 8; training session 2: n = 14; training session 3: n = 3).

The training sessions were mainly conducted according to the training manual. Deviations occurred in particular due to the pandemic-related changeover to telephone training or the online format, when for example, role plays or group work could not be carried out.

### Evaluation of the educational programme

A total of 50 GPs completed the training evaluation after the second mandatory training session. Overall, the training was positively evaluated by the participants, both for presence sessions as well as the telephone/online training. The content was deemed relevant by almost all participants and the professional level was appropriate. Most GPs felt well prepared to conduct the family conferences.

**Fig 2:**
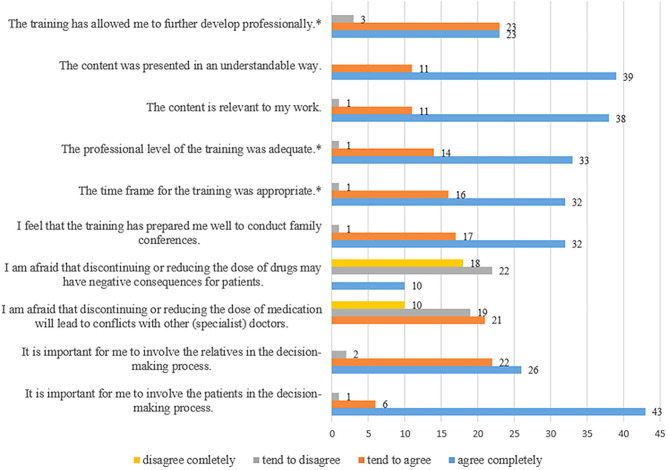
Evaluation of the educational programme by GPs. *Only valid answers counted. The overall evaluation of the training (analogous to German school grades, one to six, one being the best) was rated with a mean (SD) of 1.5 (0.5).

Involving relatives in shared decision-making was considered important by most GPs but deemed even more important for patients. In addition, there were still GPs who were afraid that deprescribing would be associated with negative consequences for patients and could lead to conflicts with other specialists (see Fig 2).

### Implementation of the family conferences

The first family conference was conducted in July 2019, the last in May 2021 by the 59 GPs. Family conferences were conducted with a total of 676 patients (first family conference: n = 244, second family conference: n = 232, third family conference: n = 200). Of the 272 patients in the intervention group, 185 patients (68%) received all three planned family conferences, 46 patients (17%) received a total of two family conferences, and 29 patients (11%) received at least one family conference. Twelve patients (4%) did not participate in a family conference at any time. The first family conference lasted on average 37 minutes (median: 30 minutes; SD: 15.57), the second family conference lasted 35 minutes (median: 30 minutes; SD: 12.94), and the third family conference lasted 32 minutes (median: 30 minutes; SD: 14.70). The duration of the family conferences ranged widely between ten and 120 minutes.

A total of 342 family conferences were conducted in the patients’ homes, 213 family conferences took place in GP practices, and 80 family conferences were held via telephone or videoconference (information is missing for a total of n = 41 family conferences).

In 410 (60.7%) family conferences at least one relative participated, both family members and representatives from the home care service attended 115 (17.0%) family conferences and in 29 (4.2%) family conferences only representatives from the home care service were involved. In a total of 120 (17.7%) family conferences, neither a family member nor a representative of the home care service participated (n = 2 missings).

Table 1 shows the medical and non-pharmacological measures and recommendations resulting from family conferences documented by the GPs. This comprised the adjustment of medication (including, e.g., dose reduction, prescription, discontinuing and change of medication) and other medical treatments or diagnostics like referrals to other specialists, flu vaccination or blood tests. Further measures and recommendations included, among others, the prescription of therapies (physiotherapy, logopaedics, or occupational therapy), advisory consultations (e.g., regarding other treatment options), encouragement of increased mobility, and the prescription of technical/care aids (e.g., walker). In the first family conference, no measures or recommendations (apart from adjustment of medication) were agreed upon for 103 patients (42%), in the second family conference this applied to 66 patients (32%), and in the third family conference to 62 patients (31%).

**Table 1:** Medical and non-pharmacological measures and recommendations resulting from family conferences.

Measures & Recommendations, n	FamCo 1 (n = 244)	FamCo 2 (n = 232)	FamCo 3 (n = 200)
Changes of medication	214	124	106
Other medical treatments/diagnostic (e.g., flu vaccination, blood test)	56	64	64
Prescription of physiotherapy, logopaedia or occupational therapy	31	46	41
Advisory consultations (e.g., other treatment options)	32	45	26
Encourage more mobility	11	26	16
Prescription of technical/care aids	14	20	10
Prescription of treatment care (changing bandages, giving injections)	18	14	8
Application (e.g., patients’ need for care assessed by the medical service of the German social care insurance)	9	18	7
Own monitoring of vital parameters & weight	14	6	6
Adaption of living space	2	8	4
Support by relatives	3	1	5
Others	13	23	19
**Total**	**427**	**385**	**312**

Values are absolute frequencies. Abbreviation: FamCo, family conference.

### Use of the pharmacological hotline

The GPs in the intervention group had the opportunity to consult an external clinical pharmacologist/pharmacist via phone or email. This was utilised eight times during the course of the study. The questions were very heterogeneous and predominantly specific to individual patients.

### Experiences of patients and relatives

A subsample of 24 patients and 18 relatives participated in the telephone interviews. The mean duration of the interviews was 22 minutes (min: 8 minutes; max: 54 minutes). The participants’ characteristics are presented in Table 2.

**Table 2:** Characteristics of patients and relatives.

Characteristics	Patients (n = 24)	Relatives (n = 18)
Age, mean (SD) [min-max], years	85.3 (4.8) [75-93]	72.5 (12.6) [57-95]
Sex, No.		
Female	19	14
Male	5	4
Care dependency category, No.^a^ (6 missings)		
None	1	6
Level 1 (Low)	2	0
Level 2	10	2
Level 3	7	6
Level 4	1	1
Level 5 (Very severe)	0	0
Form of living, No.^b^		
Flat	11	–
House	13	–
Relationship to the patient, No.^c^		
Spouse	–	6
Child	–	9
Son-/daughter-in-law	–	3

^a^Participants’ need for care (including, e.g., mobility, cognitive and communicative abilities, organising everyday life and social contact) assessed by the medical service of the German social care insurance.

^b^Information only obtained from patients.

^c^Information only obtained from relatives.

#### General experiences with family conferences.

The experiences of patients and relatives regarding the family conferences were largely positive. This was associated with a generally good relationship with the GP. A particularly positive aspect of the family conferences was that the GP took plenty of time to discuss all questions and concerns in the patient’s home.

*Yes, I thought that was good. It wasn’t bad because you could also talk about other problems and so on. I mean, when you go to the GP, for instance, they often don’t have that much time to talk about any other problems. (Interview 5 (patient): pos. 42)*

Both patients and relatives felt they had been sufficiently involved in the joint discussion with the GP during the family conferences.

#### Consideration of the preferences.

The adjustment of medication was perceived by the patients and relatives as the main focus of the family conferences, in addition to the general health and care situation. If indicated, drugs could be reduced or discontinued. In some cases, the drugs had to be restarted after patient follow-up, or no changes were made. According to the patients and relatives interviewed, they were able to contribute sufficiently to the joint discussion and the preferences were taken into account in the decision-making process. But it also became clear that patients and relatives were not often able to judge the recommendation to discontinue or reduce medication from a professional point of view and relied on the judgement of the GP.

*[…] because I always had the feeling that A) I can always ask, and B) I can also influence things. And if I had said it was too risky for me, I don’t know if he [GP] would have got his way, but I didn’t have that feeling at all. (Interview 26 (relative): pos. 39)*

However, some of the patients and relatives interviewed also reported concerns about the discontinuation of medication.

*[…] I also felt a bit helpless, I have to say, because she [GP] said we were cancelling all that, she [patient] didn’t need it any more. And I had the feeling that it was for cost reasons or something, that it was all cancelled now, and I didn’t know really know the context or couldn’t make sense of it […]. (Interview 14 (relative): pos. 46)*

In one case, a drug was continued by the patient not adhering to the agreement with the GP.

#### Lacking awareness of the intervention.

For some of the participants, the family conferences did not differ from usual home visits by GPs.

*It was so normal, like always, she [GP] asked, and I answered […]. (Interview 4 (patient): pos. 40)*

Sometimes it was apparent that the family conference itself was not perceived as an intervention, but rather the data collection as part of the COFRAIL study.

#### Pandemic-related influences.

For some participants, the family conferences took place after the onset of the SARS-CoV-2 pandemic, but neither patients nor relatives experienced any negative effects in terms of implementation.

*[…] but the doctor still kept coming to visit me at home. So it all worked out wonderfully. (Interview 5 (patient): pos. 67)*

### Experiences of general practitioners

Telephone interviews were conducted with a subsample of 23 GPs out of a total of 59 GPs in the intervention group. The interviews lasted on average 16 minutes, with the shortest being eight minutes and the longest being 34 minutes. Table 3 provides the descriptive characteristics of the participants.

**Table 3:** Characteristics of general practitioners.

Characteristics	(n = 23)
Age, mean (SD) [min-max], years	48.3 (7.8) [36-62]
Sex, No.	
Female	12
Male	11
Specialisation, No.	
General practice	14
Internal medicine	5
Internal medicine and general practice	2
Anaesthesia	1
Advanced training assistant	1
Employee status, No.	
Self-employed	21
Employed	2
Years in general practice, mean (SD) [min-max], years	12.8 (9.9) [3-35]
Practice form, No.	
Single	8
Group (≥2)	15
Practice size (Number of patients treated at least once per quarter), mean (SD) [min-max]	1,715 (1,065) [500−4,300]

#### General experiences with family conferences.

The GPs also reported predominantly good experiences with family conferences. From their perspective, the patients perceived it as particularly positive that they were cared for more intensively. Direct communication with the patients and their relatives was perceived as very helpful.

*Yes, it was definitely a good appointment to have a conversation with the family without there being an acute crisis or anything like that. Which is something that doesn’t necessarily happen in normal therapy, so I think both sides definitely benefited from having a general discussion. (GP 3: pos. 12)*

#### Involvement of patients and relatives.

The GPs reported that patients and relatives were mostly involved in the decision-making process and concerns were taken into account, and medication was not always discontinued. The relatives played a supporting role.

*But what was very good was the involvement of the relatives, which I think gave the patients more confidence that they could make a joint decision, because they had the feeling that the family was behind it, or the husband or daughter, and then of course that also made some treatment decisions easier. (GP 9: pos. 12)*

The main focus of the family conferences was on adapting the medication. In general, a positive attitude toward deprescribing was apparent among GPs. However, it was sometimes reported that patients or relatives refused to reduce the medication.

*I was actually surprised at how unwilling patients are to change anything. So the deprescribing, that’s always a good idea, but even if you have something in mind for the conference, two days later you get a call from the relative saying, no, that’s not possible because so and so. So it was actually a bit frustrating in retrospect. (GP 5: pos. 22)*

#### Usefulness of supporting components.

The deprescribing manual, used both as a decision-making and an argumentation aid in the family conferences, was considered particularly supportive.

*So I kept checking again and again. It always reassured me that I had already cancelled certain things. (GP 2: pos. 28)*

The pharmacological hotline was not often used, as little need for it was seen.

#### Barriers and facilitators for the implementation of family conferences.

The organisation and implementation of the family conferences were predominantly experienced as unproblematic, and the relatives could mostly be well integrated. In some cases, it was not possible to involve the relatives (e.g., if relatives living far away or due to lack of time). Furthermore, the scheduling was sometimes difficult.

*Yes, it didn’t always work out directly. With one patient we had a conference without the relatives, but otherwise it worked out with them. (GP 15: pos. 16)*

The time expenditure was perceived very differently. Most of the GPs interviewed considered the amount of time to be appropriate. However, some considered the implementation of family conferences in regular care to be impracticable and too time-consuming. In some cases, it was not considered necessary to conduct family conferences in the patients’ home or as comprehensively and structured as envisaged in the guideline developed for conduction of the family conference in the COFRAIL study.

*[…] Patients who normally go shopping or come to our practice. And I think the effort is simply too high, because home visits are always very time-consuming. You have the journey, you have to find a parking space, you’re simply there for longer because the patients in their familiar surroundings naturally have other issues that they want to address and then the relatives also have ten questions. (GP 9: pos. 34)*

*Not with a standardised sheet every time, so I don’t have a formal conference at home with the patients, but I have already incorporated the content into my daily practice. (GP 17: pos. 54)*

The SARS-CoV-2 pandemic was another relevant contextual factor, but the GPs, like the patients and relatives, did not experience any negative effects regarding implementation of the study intervention. In some cases, family conferences were conducted by telephone or online to avoid personal contact.

*We had family conferences under corona precautions. In other words, I also wore a gown in two cases because relatives were of a certain age and it wasn’t safe because I had to deal with potentially contaminated people on a daily basis. I always wore a mask and occasionally wore gloves, but it wasn’t really a problem. (GP 19: pos 30)*

## Discussion

The results of the process evaluation revealed that the COFRAIL intervention programme was largely implemented as intended, with adequate fidelity and intensity. The training sessions were mainly conducted according to the training manual and almost all intervention GPs completed both mandatory training courses. Slightly less than half of the GPs also participated in the third optional training. Overall, the GPs were highly satisfied with the educational programme and felt well prepared for conducting the family conferences. However, some GPs were still concerned that deprescribing might be associated with negative consequences for patients and could lead to conflicts with other specialists. This finding has previously been identified as a common barrier to deprescribing [37].

More than two-thirds of the patients received all three family conferences, and 85% of the patients received at least two family conferences that included structured medication reviews. Two earlier cRCTs investigated intervention programmes including among others GP-led medication reviews and reported that 87% and 78%, respectively, of medication reviews were conducted [38–40]. Thus, the intensity is similar to that of other studies. The first intervention programme was effective in reducing potentially inappropriate prescribing (PIP), which refers to various suboptimal prescribing practices, particularly the use of medication that implies a negative benefit-harm balance [38,39]. The latter study found that the intervention programme resulted in a small but significant reduction in the number of medications, but no significant reduction in PIP. However, the study only included patients with a much higher number of prescribed medications than in COFRAIL (≥15 vs. ≥ 5), which suggests a higher deprescribing potential [15,40,41].

In contrast, another study described an implementation failure, with only 49% of patients receiving both intended medication reviews and 30% receiving a partial review, resulting in no significant effect on either the primary trial outcome (quality of life) or the secondary outcome (number of medications prescribed) [42].

The COFRAIL intervention was somewhat unique in involving family members in the decision-making process. This involvement is crucial, as it can provide additional support and reassurance to patients during the decision-making process, potentially enhancing the acceptability and effectiveness of deprescribing efforts. In about 80% of the family conferences, at least one relative participated. To our knowledge, no interventional study aimed at improving drug safety in older adults with frailty in primary care involved relatives in the intervention programme [43].

The duration of the COFRAIL family conferences varied widely between ten and 120 minutes. It is therefore questionable to what extent the patients’ needs have always been adequately addressed and prioritised. The GPs’ documentation indicated that the focus of the first conference seemed to be clearly on the adaption of medication and in the subsequent conferences on non-pharmacological measures and recommendations as was intended in the intervention concept. As described in the published main study [15], after six months the mean number of PIMs was significantly lower in the intervention group compared to the control group (1.30 [SD: 1.05] vs. 1.71 [SD 1.25]; P = 0.04). An additional exploratory analysis found that mostly proton pump inhibitors, urate-lowering medications, statins, and oral antidiabetic agents were discontinued. After twelve months, there was no significant difference [15]. An in-depth analysis of the medication changes will be published separately. However, there was also a large proportion of patients who did not receive any non-pharmacological measures or recommendations as a result of the family conferences. This may indicate that these patients already received appropriate care or that there were some inconsistencies in the delivery of the intervention.

Overall, the family conferences were experienced very positively by the interviewed patients, their relatives and the GPs. The patients and relatives usually felt sufficiently involved in the family conferences and the decision-making process. Earlier evidence suggests that it is otherwise an important barrier to deprescribing interventions [44]. The adjustment of medication was perceived by the patients and relatives as the main focus of the family conferences, and most patients and relatives relied on the judgment of the GP. Physician trust has been described as an important factor that also may inﬂuence the extent of acceptance with regard to deprescribing [45]. However, some concerns about discontinuation of medication were also expressed.

Interestingly, it emerged during the interviews that some patients and relatives primarily perceived the data collection of the main study as part of the intervention. A previous study suggested that the intensive study data collection led to a potentially substantial bias and had the same effect in the control group as the structured medication reviews in the intervention group [46]. However, in our study, there were no significant changes regarding the main study outcomes after 12 months, neither in the intervention group nor in the control group [15].

Another component of the COFRAIL intervention was a pharmacological hotline, where the intervention GPs had the opportunity to consult an external clinical pharmacologist/pharmacist via phone or email [15]. Several studies involved pharmacists and pharmacologists in the intervention programmes aimed at improving appropriateness of medication regimes and the quantity of drugs with inconclusive results [42,47–52]. However, this supportive component was rarely utilised as the GPs expressed little need for it. Pharmacological counselling or reassurance may already have been sufficiently covered by the deprescribing manual provided. In addition, emerging questions may also have been clarified as part of the COFRAIL training programme by means of peer discussions. Regardless of this, perhaps the additional opportunity of using a pharmacological hotline could have been promoted more intensively to the GPs. Furthermore, possible barriers on the part of the GPs could be discussed in more depth during the training programme.

Overall, we identified no major barriers in the implementation of the intervention programme. Only a few GPs considered the implementation of family conferences in routine care to be impractical and too time-consuming. Involving family members or representatives of home care service in family conferences was mentioned by some GPs but was not described as a major challenge. In contrast, in quite a lot of family conferences neither a family member nor a representative of the home care service attended.

Although some GPs indicated concerns about conflict with other specialists after the educational programme, it was not reported as a specific barrier. From February 2020, the COFRAIL study was affected by the onset of the SARS-CoV-2-pandemic in Germany. As in many other studies [53,54], some procedures in the COFRAIL study had to be adapted. Thus, some training sessions and family conferences were held via telephone or online. However, from the perspective of patients, relatives and GPs this change in study procedures did not appear to disturb the process of a family conference to a relevant extent.

### Strengths and limitations

Our process evaluation was based on the recommendations for process evaluations for cluster randomised trials of complex interventions [34]. Quantitative and qualitative methods were rigorously applied at different stages of the main study. The perspectives of patients, their relatives and GPs regarding the intervention programme and its implementation were comprehensively covered. The interviews were conducted by interviewers who were not involved in the main study, reducing the likelihood of socially desirable responses from the interviewees.

Data saturation was achieved both in the interviews with GPs and in the interviews with patients and relatives. However, convenience sampling for the interviews should be considered, which might affect the transferability and credibility. It cannot be ruled out that particularly motivated GPs with a more positive attitude toward deprescribing as well as less frail, and ill, or cognitively impaired patients agreed to participate in the interviews. The characteristics of the interview samples do not support this assumption, since they are largely comparable with the overall population of the COFRAIL study. Furthermore, the interviews with patients and relatives were planned as dyad-interviews, which turned out to be difficult to realise. Relatives sometimes did not live in the same household, or the patients were willing to take part in an interview but the relatives were not, or vice versa. Therefore, the methods were adapted to the research context and the interviews were mainly conducted separately, which likely led to a different dynamic of the conversations.

Although the interviews provide some insights, it remains unclear in this process evaluation to some extent whether the documented medical and non-pharmacological measures and recommendations resulting from family conferences were actually implemented by the patients and relatives. It is also uncertain how consistent the quality of the family conferences among the GPs was, as envisaged by the training programmes. Furthermore, the process evaluation focused on the implementation of the intervention rather than on the usual care provided by the GPs in the control group. Therefore, it remains unclear whether and which recommendations were made to their patients during the study period. It cannot be ruled out that the perceived satisfaction with the increased time available with the GP, as expressed by patients in the interviews, may also reflect a bias due to the open trial design of the COFRAIL study without blinding towards the intervention. However, these process measures were not collected in the control group. Moreover, the process evaluation focused more on the process of implementing the intervention and the experiences of the target groups. It was not possible to fully understand the ineffectiveness of the COFRAIL intervention on the basis of this process evaluation, even though some relevant factors were taken into account.

## Conclusions

The COFRAIL study sought to address the significant challenges of polypharmacy and frailty in older adults by implementing a structured intervention centred on family conferences. Findings of the COFRAIL trial and this process evaluation suggest that family conferences for shared decision-making can successfully initiate the process of discontinuing medication. Despite the comprehensive nature of the intervention, however, the study did not achieve its goals for the main outcomes of reducing hospitalisations, the number of medications or PIM.

Overall, the intervention was well implemented and no major barriers were found, suggesting that the intervention may not have had enough impact to effectively influence these outcomes.

However, the process evaluation of the COFRAIL study offers important insights into the implementation and reception of the intervention, which can inform future efforts in similar contexts. The training provided to the GPs was generally well received, but some GPs expressed ongoing concerns about deprescribing, particularly regarding the potential for negative consequences for patients and conflicts with other specialists. This highlights the need for more robust support systems and further education to address these barriers.

The family conferences themselves were largely implemented as intended, with a large proportion of patients receiving all sessions. The involvement of relatives in these conferences was positively perceived by both patients and their families. Variation in the duration of the family conferences and partly a lack of non-pharmacological recommendations suggest that there may be inconsistencies in the delivery of the intervention. Some patients and relatives did not perceive the family conferences as distinct from usual care, or associated the intervention with the data collection process of the study, indicating a need for clearer communication.

The underutilisation of the pharmacological hotline suggests that this support component was not fully integrated into the intervention or perceived as valuable by the GPs. This issue could be improved by better promotion of the hotline and a more direct involvement of clinical pharmacologists in the decision-making process during family conferences in future studies.

## Supporting information

S1 Appendix Interview guide for patients and relatives. (PDF)

S2 Appendix Interview guide for general practitioner. (PDF)

S3 Appendix eTable 1. Contents and procedures of process evaluation. (PDF)
