# Supplementary material for: Complex intervention programme to improve patient safety and facilitate deprescribing in frail older patients living at home (COFRAIL): A process evaluation of a cluster randomised controlled trial
Source: PLoS One. 2026 Jul 8;21(7):e0350664. doi: 10.1371/journal.pone.0350664 (PMC13345250; doi:10.1371/journal.pone.0350664)
Supplement: S1 Appendix — (PDF) [file pone.0350664.s001.pdf]

Telephone interview with general practitioners

Introduction

First of all, thank you very much for agreeing to take part in this telephone interview. In the interview, we would like to talk about your experiences with the COFRAIL study and the family conferences.

Procedure

Our telephone interview will be digitally recorded and will last approximately 15 minutes. All your details will be treated with absolute confidentiality. During the subsequent data analysis, your details will be pseudonymised, i.e. no names or initials will be used.
If you agree, I would like to record this interview.

→Start recording

Do you agree to this?

- *Obtain feedback*

Do you have any questions?

- *Obtain feedback*

Interview guide

Introductory question

Please start by describing in general terms how you think the family conferences went?

Procedure of the family conference

1. How many patients did you care for during the study for whom family conferences were also held?
2. Who was present at the family conferences?
   →Were they always the same contact persons or relatives?
3. Did you feel that all participants were sufficiently involved in the family conference? If not, could you please elaborate on this?
4. Is the relative also receiving treatment from you? If so, did this influence your actions?
5. What was the main focus or content of the family conferences?
   →What agreements/goals did you agree with your patient and relatives?
   →Were there any other goals/recommendations apart from adjusting the medication?
6. In your opinion, were the agreements made sufficient or appropriate? If not, what else should have been agreed in your opinion?

7. Were there differences of opinion between you and your patient or relatives regarding the discontinuation of medication or a dose reduction? If so, could you please explain this in more detail?
8. Were there any uncertainties on your part regarding the agreed goals? If yes, please describe these in more detail!
9. Were you able to understand to what extent the patients implemented the agreements or recommendations?
10. Were there any factors that negatively influenced the course of the family conference? If so, what were they?
    →Did you conduct three family conferences with all patients as planned?
    →Did the corona situation have an influence on the conduct of the family conferences or were they all prior to this? If yes, please specify.

Usefulness of the family conference guide

11. Did you find the guide helpful in organising the family conference? If not, why not?
12. Were you able to implement the contents of the family conference guide? If not, why not?
13. Were there any deviations from the guidelines? If yes, why?

Usefulness of the 'deprescribing manual'

14. Did you find the 'deprescribing manual' useful as a support for stopping medication? Did you use it to prepare for family conferences? If not, why not?
15. Which recommendations from the guide did you implement? Which recommendations do you find particularly helpful and would you adopt in your daily practice? Why?
16. Which recommendations from the guide have you not implemented and would you not implement in your everyday practice? Why?
17. Have you used the pharmacological hotline? And if not, why?
18. What is your general attitude towards deprescribing? Have you changed your attitude in this regard in the course of this study? And if yes/no, why?

Effort required to conduct the family conference

19. How much time did you need to prepare the family conferences?
20. How much time did you spend on the follow-up of the family conference? Please think about all the activities that you had to carry out after the family conference, such as the time required for documentation or arranging follow-up appointments (if not already taken into account in the family conference).
21. How many follow-up checks (monitoring) took place on average and how long did these follow-up checks take on average?
22. What other activities were required after the family conference (e.g. contact with the care service)?
23. How do you estimate the overall effort required to organise a family conference, including the preparation and follow-up work?

Need for adjustment/change

24. Were there any other problems with the organisation or the preparation and follow-up of the family conference? If yes, please describe these in more detail.
25. What do you think should be done differently or improved?
26. Will you continue to use the family conference format in your practice outside of the study in the future?
27. In your opinion, can the family conference format be implemented in standard care?
28. Are there other areas where you would find the family conference format suitable (e.g. oncology or palliative care, people with dementia)?
29. Is there anything else you would like to tell us?

Farewell

Thank you for taking part in our telephone survey and thank you for your valuable feedback!
