# Supplementary material for: Complex intervention programme to improve patient safety and facilitate deprescribing in frail older patients living at home (COFRAIL): A process evaluation of a cluster randomised controlled trial
Source: PLoS One. 2026 Jul 8;21(7):e0350664. doi: 10.1371/journal.pone.0350664 (PMC13345250; doi:10.1371/journal.pone.0350664)
Supplement: S2 Appendix — (PDF) [file pone.0350664.s002.pdf]

cofrail

Telephone interview with patients/relatives

Introduction

First of all, thank you very much for agreeing to take part in this interview. We would like to use the interview to find out how you feel the discussions with your family doctor went as part of our COFRAIL study and what your experiences were.

Procedure

Our telephone interview today will last about 15-30 minutes. All your details will be treated with absolute confidentiality. Your details will be pseudonymised during the subsequent data analysis, i.e. no names or initials will be used.

If you agree, I would like to record this interview.

→start recording

Do you agree to this?

- *Obtain feedback*

We have already received your signed written informed consent for participation.

Do you have any questions?

- *Obtain feedback*

Interview guide

Introductory question

Please start by describing in general terms how you think the study and the family conferences **(discussion with GP)** went?

Procedure of the family conference

1. How many family conferences were held with your relative as part of the study?
2. Were you present at all appointments?
3. Were other people involved in the conversation (e.g. other relatives, care service)?
4. What was the content of the discussions?

Medication

5. Did you talk about medication? Other options: Did you discuss other treatment or care options (e.g. PT, spa treatment)?
6. Did you have the feeling before the family conferences that you were taking a lot of medication?
7. Did you feel comfortable with the number of medications you were taking before the family conferences? If not, why?
8. Did you wish you could stop taking one or more medications? If so, what was the reason you did not?
9. Did you discontinue medication in the family conferences?
10. Did you have any concerns about stopping? If so, was the doctor able to allay your fears?
11. Did you feel that you had side effects from the medication that got better after stopping?
12. Did taking the medication become easier for you after the family conferences?
13. Do you now have more clarity about what you are taking each medication for than before? Why?
14. Were you satisfied with the follow-up after discontinuation? If not, why?
15. To what extent was the information provided by your relative's GP clear to you?
16. Were all important topics discussed? If not, where would you have liked more information?
17. Did you feel that you as a **patient/relative** were sufficiently involved in the family conference?
    - **a. Was your relative able to participate?**
18. To what extent did you have sufficient opportunity to ask questions?
19. Would you like to have further discussions with your relative's family doctor in this form in the future?
20. What agreements/goals were agreed with your relative's GP? möglichst Please specify
    1. 21 In your opinion, to what extent were there concerns about implementing these agreements/goals?
21. Did you feel that your wishes, ideas or concerns or those of your relative were sufficiently taken into account by your family doctor?

Experiences with the family conference

22. What expectations did you have of the family conference beforehand and to what extent were these fulfilled?
23. Did you have any questions or uncertainties following the family conference? If so, which ones?
24. How do you rate the overall approach of the family conference?
25. Did you find the family conference different from the usual home visits by your family doctor?
26. What do you think should be done differently or improved?
27. To what extent has corona affected the organisation of the family conferences?
28. Were the family conferences held online? *Further option: When did the family doctor consultations take place? Before or after Corona?*
29. Were there any other effects of the pandemic for you and your relative? *Further option: Experiencing and dealing with the coronavirus pandemic?*

30. Is there anything else you would like to tell us? *Further option: Are there things that have not yet been discussed or that you are still missing?*

Demographic information

Finally, I have a few questions about you personally.

31. How old are you?
32. Do you live in an urban or rural area?
33. Do you live in a house or flat?
34. What is your relationship to your relative (e.g. spouse, son/daughter, etc.)?
35. Do you live in the same household as your relative?
36. Do you have a care degree yourself? *(only relatives)*
37. Are you being treated by the same GP? *(only relatives)*
38. Who is caring for your relative (e.g. is a care service involved, other relatives)?

Farewell

Thank you for taking part in our telephone survey and thank you for your valuable feedback!
