# Supplementary material for: Complex intervention programme to improve patient safety and facilitate deprescribing in frail older patients living at home (COFRAIL): A process evaluation of a cluster randomised controlled trial
Source: PLoS One. 2026 Jul 8;21(7):e0350664. doi: 10.1371/journal.pone.0350664 (PMC13345250; doi:10.1371/journal.pone.0350664)
Supplement: S3 Appendix — (PDF) [file pone.0350664.s003.pdf]

eTable 1. Contents and procedures of process evaluation.

**Focus**	**Data collection methods, target groups & measurement points**
Feasibility of the intervention (piloting of family conferences)	Semi-structured telephone interview, with 2 physicians/region and with patients and relatives, prior to t_0
Recruitment procedure of physicians and patients	Protocol/region, prior to t_0
Reasons for non-participation or drop-out	Structured inquiry and documentation, t_0-t_2
Implementation of the intervention (delivery to clusters)	Mandatory educational sessions: Structured protocol of each educational session, at t_0 (immediately after the educational intervention)
	Use of facultative educational session: Standardised documentation, at t_0
	Use of individual medication reviews offered by a pharmacologist/pharmacist: Standardised documentation, t_0-t_2
Evaluation of training (satisfaction with the training, attitudes regarding deprescribing, acceptance, self-efficacy, expectations)	Standardised questionnaire, all participants of the educational programme, at t_0 (after the second educational session)
Implementation of the intervention (delivery to individuals) (acceptance, contents, duration, practicability, need for adaption)	Semi-structured protocols → evaluation of family conferences, all physicians, at t_0, after 3 and 9 months (immediately after family conferences)
Experiences of families (e.g. consideration of preferences; changes in physician-patient-communication; shared decision-making; barriers and facilitators)	Guideline-based telephone interviews, 10 patient-relative-dyads/region, after 9 months (immediately after the last family conference)
Experiences of physicians (e.g. attitudes regarding intervention; shared decision-making; changes in physician-patient-communication; barriers and facilitators)	Guideline-based telephone interviews, convenience sample of 10 physicians/region, at t_2

Measurement points: t_0=baseline, t_1=after 6 months, t_2=after 12 months.
